# Comparative study of an ai-based visual psychophysiological analysis platform and self-report scales for screening depression and anxiety: a single-center prospective diagnostic study

**DOI:** 10.3389/fpsyt.2026.1729303

**Published:** 2026-04-01

**Authors:** Hongmei Zhu, Hongwen You, Yuting Nie, Yangfan Sun, Liyan Duan, Peiyu Yan, Yingqi Chen, Ming Zhou

**Affiliations:** 1 Shenzhen Luohu Maternity and Children Health Care Hospital, Shenzhen, China; 2 Institute of Nanoscience and Applications, Southern University of Science and Technology, Shenzhen, China; 3 Shenzhen Zhichan Artificial Intelligence Technology Co., Ltd., Shenzhen, China

**Keywords:** anxiety disorder, artificial intelligence, depressive disorder, digital phenotype, psychophysiological assessment

## Abstract

**Background:**

Depression and anxiety are among the most prevalent psychiatric disorders in clinical practice. Their high comorbidity and the inherent subjectivity of self-report screening tools have motivated efforts to identify objective, physiology-based digital phenotypes.

**Objectives:**

To rigorously evaluate the diagnostic performance of an artificial intelligence visual analysis platform based on head–neck micro-vibration signals for screening depression and anxiety, to compare its differences and complementarities with traditional self-report scales, and to develop and explore the potential utility of a combined “AI broad screening + scale refinement” approach.

**Methods:**

We conducted a single-center prospective diagnostic study enrolling 98 outpatients. A psychiatrist-administered structured interview grounded in DSM-5 served as the clinical diagnosis. All participants completed Self-Rating Depression Scale (SDS) and Self-Rating Anxiety Scale (SAS) assessments in parallel with testing by the AI psychophysiological analysis system. We constructed confusion matrices, calculated F1 scores, and generated receiver operating characteristic curves and decision curve analyses to quantify and compare the screening and stratification performance of each tool and of the combined models.

**Results:**

For depression-risk screening, the AI tool demonstrated very high sensitivity (95.9%), exceeding that of the SDS (83.6%). The combined “AI + SDS” model further increased sensitivity to 98.6%, demonstrating a minimized false-negative rate in this cohort. For anxiety, integrating AI with the SAS increased recall by 50.0% (to 69.2%) and improved the F1 score by 25.4%. In-depth analyses revealed that the AI system was particularly effective at identifying “silent patients” with alexithymia or prominent somatization, whereas the scales aligned more closely with clinical judgment for fine-grained severity grading. ROC and decision curve analyses consistently showed that the combined “AI + SDS/SAS” model achieved the best overall discrimination and greatest net clinical benefit.

**Conclusions:**

This study demonstrates that an AI tool based on head–neck micro-vibration signals can serve as a high-sensitivity, objective sentinel, mitigating the risk of missed cases associated with subjective self-report scales in specific populations. AI and self-report measures capture complementary facets of psychopathology. A tiered workflow of “AI broad screening + scale refinement” may constitutes a translationally promising paradigm to facilitate earlier, more objective, and efficient screening and to support more precise interventions in psychiatric disorders.

## 1 Introduction

Depressive and anxiety disorders are leading mental health conditions contributing to the global burden of disease. In clinical practice they are not only highly prevalent but also frequently co-occur (1, 2). As delineated in the *Diagnostic and Statistical Manual of Mental Disorders, Fifth Edition* (DSM-5), depressive disorders are characterized by persistently low mood and diminished interest or pleasure, accompanied by a range of cognitive and physiological alterations. Anxiety disorders, by contrast, are typified by excessive, difficult-to-control worry together with somatic tension and autonomic symptoms. These conditions often intersect, markedly worsening overall functional impairment and substantially increasing suicide risk. Epidemiological surveys indicate a lifetime prevalence of approximately 10–15% for major depressive disorder and over 20% for anxiety disorders, with comorbidity rates approaching 50%. The resulting burden imposes profound suffering on patients and families and constitutes a substantial socioeconomic challenge (3).

Despite the clear framework provided by international diagnostic standards such as DSM-5, substantial challenges persist in real-world use, particularly in large-scale population screening and primary care settings. The structured clinical interview entails complex procedures, is time-consuming, and requires highly trained evaluators, limiting its feasibility in resource-constrained environments (3, 4). By contrast, widely used self-report instruments, such as the Self-Rating Depression Scale (SDS) and Self-Rating Anxiety Scale (SAS), have become mainstays of clinical screening and epidemiologic surveys because they are convenient and inexpensive (1, 2). However, these tools fundamentally depend on respondents’ introspection and self-report, rendering them vulnerable to response biases, social desirability, stigma, transient psychological states, and cultural context. As a result, both sensitivity and specificity are subject to a practical ceiling effect. Consequently, the current ecosystem of clinical tools has yet to achieve an optimal balance between diagnostic accuracy and scalability.

Researchers have recently explored a new field called digital phenotyping to address this bottleneck. Its goal is to identify biobehavioral markers of specific psychological states. This is done by passively and continuously collecting objective data on individuals’ behavior, speech, motor function, and physiology. This data is gathered from smartphones, wearables, and video analytics in natural settings. Within this line of work, vibration imaging grounded in the vestibulo-emotional reflex theory provides a distinctive and promising entry point. The theory posits that emotions and psychological states, modulated by the vestibular system, are externally manifested as persistent, minute, involuntary movements of the head and neck. The micro-vibration typically falls within physiologically relevant frequencies of 0.1–10 Hz, with amplitudes of 10–1000 micrometers; although imperceptible to the naked eye, they can be precisely captured and quantified using high-frame-rate imaging and computer-vision algorithms. Preliminary evidence indicates that, relative to healthy controls, patients with depression or anxiety exhibit statistically significant differences in features of head micro-movements—such as mean energy, frequency distribution, and signal complexity—suggesting that this objective signal may serve as a potential biomarker for psychiatric risk assessment (5).

However, traditional vibration-imaging systems have typically relied on hand-crafted features (e.g., frame-to-frame difference statistics) and empirically set fixed thresholds to infer psychological states. Such approaches often fall short in diagnostic validity and cross-context robustness when confronted with individual heterogeneity, environmental variation, and complex comorbidity (6). To address these limitations, the present study builds on the independently developed *Psychological State AI Visual Analysis and Assessment Platform V1.0* by *Shenzhen Zhichan Artificial Intelligence Technology Co., Ltd.*, proposing an improved deep-learning based analytic method. Using a high-resolution camera to acquire video of the head–neck region, the method fuses the spatial feature-extraction strengths of convolutional neural networks with the temporal-dependency modeling of long short-term memory networks to perform end-to-end deep representation learning of micro-vibration signals. The model outputs continuous risk probabilities for depression (labeled AI-Dep) and anxiety (labeled AI-.

Anx) (7).

The study design was closely aligned with DSM-5 diagnostic logic, mapping AI-derived continuous risk probabilities onto three potential actionable tiers:

(1) Asymptomatic or mild symptoms: recommend routine follow-up;
(2) Moderate symptoms: recommend psychological adjustment or specialty follow-up;
(3) Severe symptoms: strongly recommend referral to psychiatric specialty care.

This stratification strategy not only accords with internationally accepted clinical practice standards but also substantially enhances the interpretability and operational utility of the findings in real-world settings. The study was conducted at *Shenzhen Luohu Maternal and Child Health Hospital* (ethics approval No. 1022024122007702), and all procedures were reviewed by the institutional ethics committee. All enrolled participants first underwent diagnostic confirmation by a board-certified psychiatrist at or above the associate chief physician level, after which they completed, in an independent setting, the AI tool assessment followed by the SDS and SAS to ensure independence of data collection and adherence to blinding principles.

Methodologically, this study aimed to systematically compare the AI tool with traditional scales by constructing detailed confusion matrices and computing composite performance metrics such as the F1 score, focusing on differences in risk detection (i.e., sensitivity) and classification precision. Furthermore, we developed a dual-axis predictive model to explore and quantify the potential diagnostic gain achieved by joint use of the AI tool and traditional scales (8).

In sum, the core objectives of this study are threefold:

1. Empirically evaluate the objective diagnostic accuracy of an AI visual analysis platform based on head micro-vibration analysis in detecting depression and anxiety disorders.
2. Delineate and compare the AI assessment paradigm and traditional self-report scales with respect to screening sensitivity, specificity, and the grading of symptom severity.
3. Prospectively explore a collaborative clinical model in which AI conducts broad initial screening and scales provide fine-grained stratification, with the aim of furnishing preliminary evidence for a future screening and early-intervention system that could be more efficient, more objective, and scalable to large populations.

## 2 Methods

### 2.1 Theoretical framework and algorithmic innovation

The core technical foundation of this study is the theoretical framework of head micro-movement psychology. This framework posits that, in a natural resting state, the human head is not absolutely motionless but under joint modulation by the vestibular system and its neurally connected limbic system, exhibits continuous micrometer-scale movement. This movement pattern, referred to as vibration-imaging head micro-movements, has frequencies primarily within the physiologically relevant 0.1–10 Hz band and amplitudes of approximately 10–1000 μm. Importantly, this involuntary motion is driven by a vestibulo-emotional reflex mechanism, and its dynamic characteristics (e.g., frequency distribution, amplitude stability, and power spectral density) can reflect an individual’s internal emotional arousal level, psychological stress state, and emotion-regulation capacity (9, 10). In psychologically healthy states, the micro-vibration signal exhibits a degree of regularity and stability; in pathological states such as depression or anxiety, dysfunction of the vestibulo-emotional pathway may lead to marked variations in both frequency- and time-domain features, for example, increased low-frequency energy and reduced signal complexity.

Traditional vibration-imaging systems approximate displacement by computing frame-to-frame differences in a video sequence and, within a specified time window, compute the mean absolute deviation of pixel-intensity changes to generate an amplitude map A (x, y), defined as:


$$\text{A}(\text{x},\text{y})=\frac{1}{\text{N}}\sum_{\text{i}=1}^{\text{N}}\left|{\text{U}}_{\text{x},\text{y},\text{i}}-{\bar{\text{U}}}_{\text{x},\text{y}}\right|$$

Where $U_{x,y,i}$ denotes the intensity of pixel (x, y) in the i-th frame, ${\bar{U}}_{x,y}$ is the mean intensity of that pixel within the time window, and *N* is the window length (11). In parallel, by applying a short-time Fourier transform (STFT) to the intensity time series $s_{x,y}(t)$ at each pixel, we extract its dominant frequency component to generate a “frequency map,” F (x, y):


$$F(x,y)=\arg\underset{f\in B}{\max}|\text{STFT}\{s_{x,y}(t)\}(f)|$$

Here, f∈B denotes the prespecified psychologically meaningful frequency band (0.1–10 Hz). Building on empirical statistical features of these images (e.g., regional mean, variance, and entropy), the system then applies predefined linear or nonlinear mapping models to produce scores on psychological dimensions such as tension, inhibition, and stability (12).

Despite offering a degree of physical interpretability, this traditional approach depends heavily on the completeness of hand-crafted feature design and the presumed universality of heuristic thresholds. It is sensitive to environmental noise, individual heterogeneity, and nonstandard acquisition conditions, which limits its generalizability.

**Figure 1:**
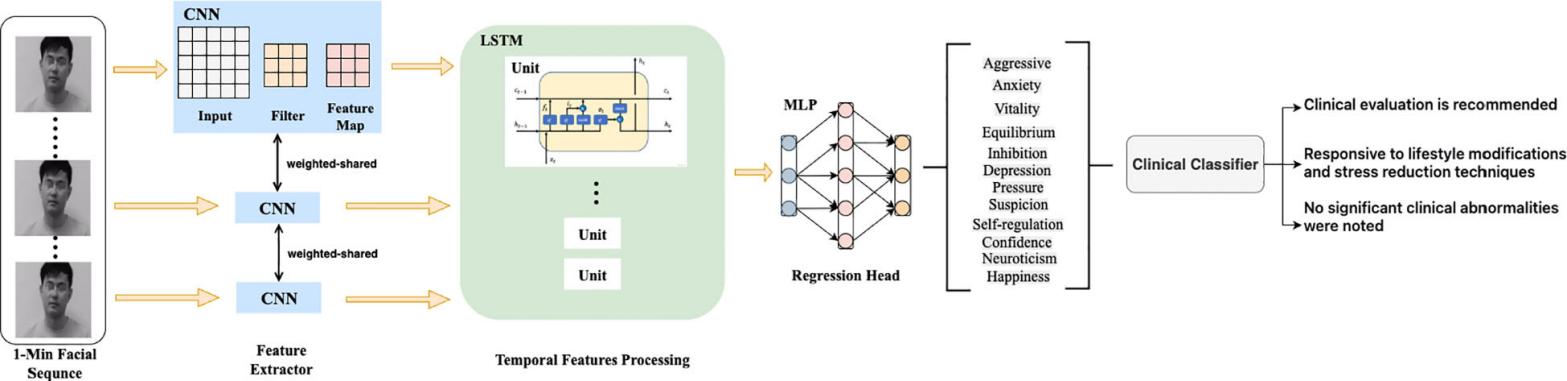
Facial sequence-based psychological state assessment framework using CNN–LSTM architecture.

To fundamentally enhance model robustness and discriminative performance, we designed and implemented an improved approach that deeply integrates computer vision with sequence learning (13–16). Workflow of the improved method, as **Figure 1** shown.

- Spatial encoding: A lightweight CNN backbone (ResNet-18) encodes each time slice t of facial video into a low-dimensional, high-level feature vector $z_{t}\in R^{d}$, aiming to capture local spatial patterns of micro-movements in the facial and cervical regions (17).
- Temporal modeling: The sequence of feature vectors $\left\{z_{1},z_{2},\ldots ,z_{T}\right\}$ is fed into a bidirectional LSTM. The BiLSTM learns long-range dependencies in both the forward and backward directions. Its final hidden state (h) aggregates global context over the entire time window, enabling representation of stable, low-frequency psychophysiological patterns while retaining transient features that are sensitive to momentary affective fluctuations (17, 18).
- Multi-task prediction heads: The temporal representation h is passed to two parallel prediction heads:
- Classification head: A fully connected layer followed by a sigmoid activation function outputs risk probabilities for depression (AI-Dep) and anxiety (AI-Anx):

$$p_{\text{dep}}=\sigma W_{\text{dp}h+b_{\text{dep}}},\;p_{\text{anx}}=\sigma W_{\text{ax}h+b_{\text{anx}}}$$

σ Regression Head: A separate fully connected layer outputs continuous severity scores for each condition, providing complementary dimensional assessment alongside categorical risk probabilities.

To translate model outputs into clinically actionable insights, we applied a multi-stage post-processing pipeline. Continuous probability scores were calibrated using temperature scaling to improve reliability and cross-participant consistency (19). Subsequently, two optimal thresholds were determined on the validation set based on the Youden index and clinical cost-benefit considerations, stratifying patients into three distinct risk tiers: (1) asymptomatic or mild, (2) moderate, and (3) severe, which are which aligned with the DSM-5 diagnostic framework. This tiered system supports clinical inference by differentiating cases responsive to lifestyle modifications and stress reduction from those requiring more intensive intervention, with the model ultimately aiding in preliminary clinical evaluation where no significant clinical abnormalities were initially noted.

### 2.2 Study design and participants

This single-center, prospective diagnostic accuracy study was conducted in accordance with the *Standards for Reporting Diagnostic Accuracy Studies* (STARD 2015) (20). The study took place from Feb. 2025 to Sep. 2025 in the outpatient Clinical Psychology Department of Shenzhen Luohu Maternal and Child Health Hospital. The protocol was reviewed and approved by the hospital’s medical ethics committee (approval No. 1022024122007702). Written informed consent was obtained from all participants or their legal guardians prior to enrollment.

A total of 98 outpatients were recruited by convenience sampling. Inclusion criteria were: (1) age 12–65 years; (2) adequate reading comprehension or the ability to understand scale items with assistance from study staff; and (3) clear consciousness and ability to complete an approximately 20-minutes assessment procedure. Exclusion criteria were designed to remove factors that could confound micro-vibration signals or affect diagnosis, including: (a) a current DSM-5 diagnosis of a schizophrenia spectrum disorder, bipolar disorder, or other severe mental illness; (b) known organic brain disease, neurodegenerative disease, or a history of traumatic brain injury; (c) moderate or greater substance use disorder (excluding nicotine); and (d) inability to maintain at least 1 minute of basic stillness during video acquisition (e.g., severe akathisia, Parkinson disease tremor).

**Figure 2:**
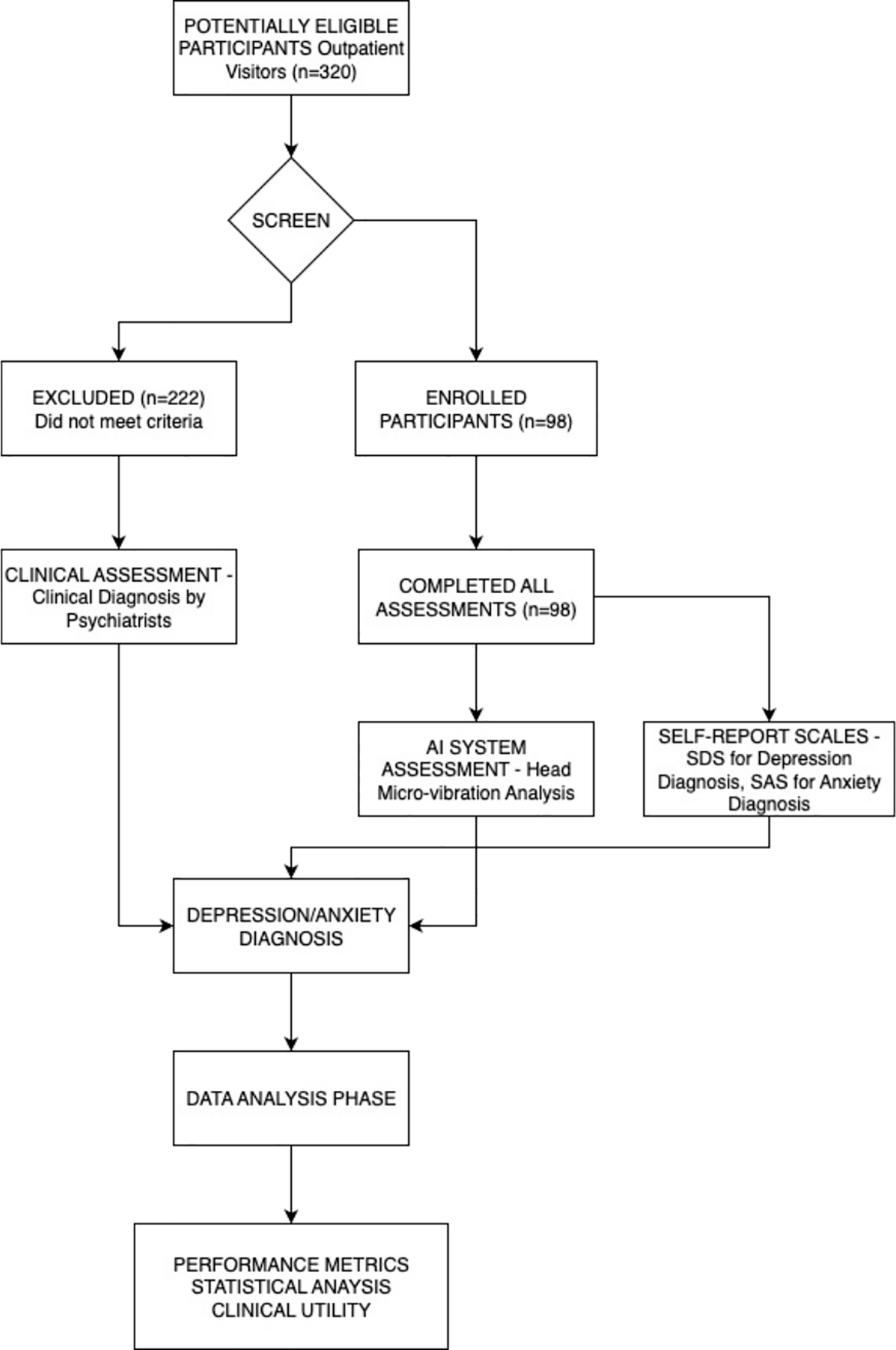
Flow diagram illustrates participant screening, enrollment, and assessment process.

Among the 98 participants who completed all assessments, the age distribution was as follows: adolescents aged 12–18 years constituted the largest subgroup at 76.5% (n=75); young adults aged 19–35 years accounted for 12.2% (n=12); middle-aged adults aged 36–55 years accounted for 10.2% (n=10); and those aged 55–65 years comprised 1.0% (n=1). This sample structure reflects the service population of the study setting (a maternal and child health hospital), as **Figure 2** shown.

### 2.3 Experimental procedure and assessment instruments

All eligible participants who provided written informed consent completed the following three core assessments in a fixed order to minimize inter-assessment interference:

1. Clinical diagnosis: Two psychiatrists at or above the associate chief physician level independently conducted semi-structured clinical interviews based on DSM-5 diagnostic criteria. The interviews covered current symptoms, illness course, functional impairment, and exclusion criteria. The two physicians’ diagnostic conclusions were checked for concordance. In cases of disagreement, a more senior chief psychiatrist was invited to adjudicate under blinded conditions, and the adjudication served as the final clinical diagnosis (21).

For subsequent quantitative analyses, the physicians’ narrative diagnostic conclusions were transformed—according to severity-defining keywords—into a computable two-axis quantitative model with separate 1–5 ratings for depression severity and anxiety severity, as shown in **Table 1**. These quantitative ratings were used as the ground truth for all subsequent evaluations of model performance (22).

**Table 1:** Clinical diagnosis dual-axis quantification model: clinical diagnosis severity grading.

Score	Severity	Example diagnostic keywords (Clini-cal interpretability)
5	Most Severe/with Psychotic Features	Severe depression with psychotic symp-toms, mood disorder, phantosmia, suicidal ideation; panic attacks
4	Severe	Depressive episode, mixed anxiety-depressive disorder; severe anxiety
3	Moderate	Depressive state, anxious depressive state, emotional and behavioral disorders, adjust- ment disorder
2	Mild	Sleep disorder, emotional disorders in chil-dren; mild anxiety
1	Subclinical/No Obvious Pathology	Psychological consultation, no significant ab-normality

2. Self-report scale assessment: Participants completed the Chinese versions of the SDS and SAS in a quiet, private room. The SDS comprises 20 items rated on a 4-point scale (1–4), with 10 reverse-scored items; a standard score is obtained by multiplying the raw total by 1.25. According to Chinese norms, a standard score <53 indicates no depression, 53–62 mild depression, 63–72 moderate to severe depression, and >72 severe depression. The SAS likewise comprises 20 items rated on a 4-point scale, with 5 reverse-scored items, and yields a standard score. By Chinese norms, a standard score <50 indicates no anxiety, 50–59 mild anxiety, 60–69 moderate anxiety, and ≥70 severe anxiety (23).

3. Assessment by the AI psychophysiological analysis system: After completing the scales, participants moved to a standardized video acquisition environment with uniform lighting (avoiding backlighting), a simple background, and minimal ambient noise. Seated at a fixed position approximately 0.8–1.0 m from the camera, participants were instructed and supervised by uniformly trained research staff (with backgrounds in applied psychology). Using standardized instructions, staff asked participants to look naturally at the front-facing camera and keep the head relaxed and still for ~1 minute, avoiding speech and large facial expressions. The system automatically recorded video and performed on-device analysis in real time. The entire procedure was controlled by the AI psychophysiological analysis system software, which implements the aforementioned deep-learning model and automatically outputs continuous risk probabilities (0–1) for depression (AI-Dep) and anxiety (AI-Anx). This assessment was conducted independently of, and under blinded conditions relative to, the clinical diagnosis and the scale assessments.

### 2.4 Statistical analysis and performance evaluation

All data cleaning, management, and statistical analyses were conducted by Python 3.8. The primary libraries included: pandas and numpy for data preprocessing, scipy for descriptive statistics and between-group comparisons, and scikit-learn for building classification models, computing performance metrics, and plotting ROC curves.

Performance metrics included:

(1) Sensitivity and specificity: Assess the model’s ability to correctly identify true cases and to correctly exclude non-cases, respectively.
(2) Precision and F1 score: Precision quantifies the accuracy of positive predictions; the F1 score—the harmonic mean of sensitivity (recall) and precision—provides a balanced measure suitable for imbalanced data.
(3) Area under the receiver operating characteristic curve (AUC): Measures the model’s overall binary discrimination across decision thresholds and serves as a key evaluation metric.
(4) Confusion matrix: Provides a concise summary of classification outcomes across classes and supports computation of the above metrics as well as error-pattern analysis.

Confidence intervals for AUC, sensitivity, and specificity were estimated via Bootstrap resampling with 1,000 repetitions. Probability calibration was evaluated using the Brier score and calibration curves. All statistical tests were two-sided, with the significance level α was set to 0.05 (24, 25).

## 3 Results

### 3.1 Analysis of depression dimensions

#### 3.1.1 Performance in severity grading

In the task of classifying depression severity into three tiers: “asymptomatic/mild,” “adjustment or follow-up needed,” and “referral to specialty care recommended,” the traditional SDS outperformed the AI tool. As shown in **Table 2**, the SDS achieved an F1 score of 0.389 against the clinical diagnosis, which was higher than the AI-Dep score of 0.304. The same pattern held for precision and recall. These findings suggest that the SDS aligns more closely with clinicians’ reasoning when assessing symptom severity. This is likely because the SDS items directly correspond to core DSM-5 domains (e.g., affective, cognitive, and somatic) (25).

**Table 2:** Comparison of three-category model performance for depression dimension.

Assessment model	F1 value	Precision	Recall
SDS vs. Gold Standard	**0.389**	**0.490**	**0.452**
AI_Dep vs. Gold Standard	0.304	0.336	0.339

Bold values indicate the best result for the metric.

#### 3.1.2 Performance in risk screening

We simplified the task to a binary screen: high-risk depression (composite score ≥ 3) versus non-high-risk cases. In this context, the advantage of the AI tool was notable. As shown in **Table 3**, AI-Dep achieved a sensitivity (recall) of 95.9%, meaning it identified more than 95% of patients confirmed by the clinical diagnosis. This performance was notably superior to the 83.6% sensitivity of the SDS. Importantly, when AI-Dep and SDS were combined under a logical OR rule (i.e., the joint model is positive if either tool is positive), screening sensitivity increased further to 98.6%, significantly reducing the rate of missed diagnoses within this sample. Although the specificity of AI-Dep (4.0%) was much lower than that of the SDS (44.0%), indicating a higher false-positive rate, such high sensitivity could be advantageous for initial screening prioritization, where minimizing false negatives is the primary objective. The joint model also yielded the highest F1 score (0.847) among the three, indicating the best overall balance of performance.

**Table 3:** Binary classification model performance and combined gain for depression dimension.

Assessment model	F1 Value	Precision	Recall	Gain effect
SDS	0.813	**0.792**	0.836	–
AI_Dep	0.838	0.745	0.959	–
SDS + AI_Dep	**0.847**	0.742	**0.986**	F1 ↑ 4.1%, Recall ↑ 18%

Bold values indicate the best result for the metric.

#### 3.1.3 ROC curve analysis

**Figure 3:**
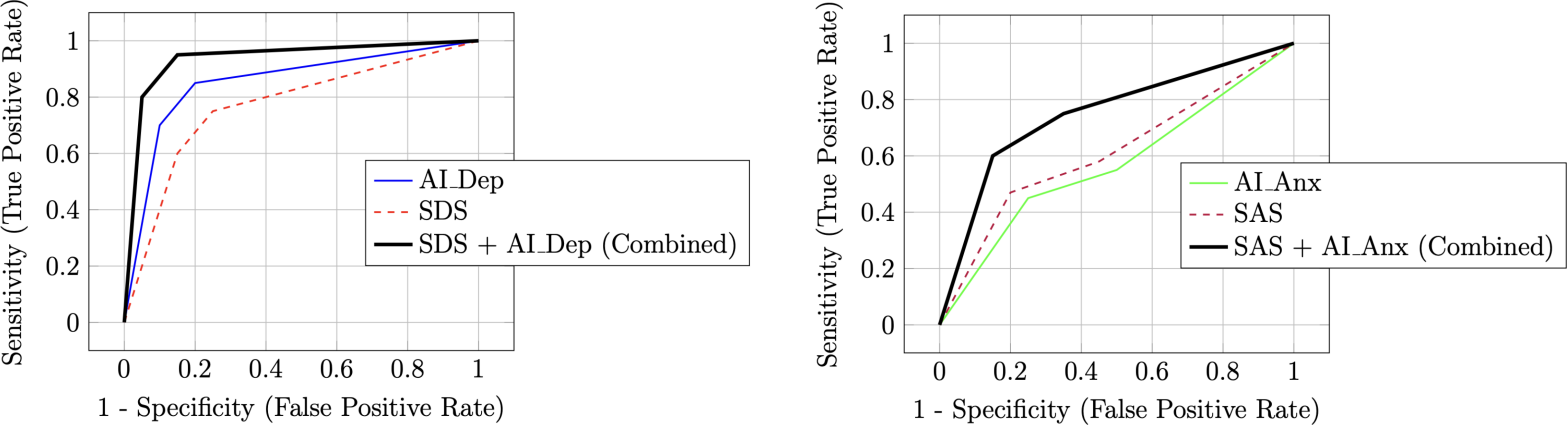
Depression and Anxiety ROC Curves (Left and Right). (Left) Depression ROC: AI Dep shows the highest AUC, and the combined SDS plus AI Dep model improves specificity at high sensitivity versus AI Dep alone. (Right) Anxiety ROC: the combined model achieves a higher AUC than either single model, indicating complementary gains..

The ROC curves (as shown in **Figure 3** (left)) provide a clear and compelling visualization of these findings. The ROC curve for AI-Dep lies closer to the upper-left corner across the full range, and its AUC is significantly greater than that of the SDS, indicating superior overall discrimination between depressed and nondepressed individuals (26). The ROC curve for the combined SDS + AI-Dep model lies above the single-model curves across most threshold values, particularly in the high-sensitivity region. This illustrates the diagnostic gain of the joint strategy, suggesting improved overall performance while maintaining high sensitivity (27).

#### 3.1.4 Confusion matrix analysis and clinical interpretation

An in-depth examination of the confusion matrix (reconstructed from **Table 3**) clarifies the sources of performance differences. AI-Dep mislabeled 24 gold-standard non-high-risk individuals as positive (false positives), compared with 14 for the SDS, accounting for AI-Dep’s lower specificity. By contrast, for true positives, AI-Dep correctly identified 70 patients, whereas the SDS identified 61.

The key clinical finding is as follows: among 73 patients classified as moderate-to-high risk by the clinical diagnosis, 11 (15.1%) were uniquely detected by AI-Dep but were negative on the SDS. A retrospective review of these 11 patients’ clinical records indicated one or more of the following features: (i) absence of a typical complaint of low mood; (ii) restricted or flat affect; (iii) pronounced alexithymia with difficulty recognizing and describing emotions; and (iv) denial or minimization of symptom severity despite objective functional impairment. This subgroup corresponds to the “silent patient” or “atypical depression” population that is most easily missed by self-report instruments reliant on subjective disclosure (28). By capturing micro-motor changes potentially mediated by the vestibulo-emotional pathway, the AI tool offers an objective approach to early detection in this group, highlighting its potential utility (29).

### 3.2 Analysis of the anxiety dimension

#### 3.2.1 Performance in severity grading

In the three-class anxiety severity task, performance was suboptimal for both AI-Anx and SAS, with F1 scores below 0.32 and no statistically significant difference between the two (**Table 4**). This likely reflects the relatively low proportion of patients whom physicians rated as having moderate-or-greater anxiety severity in our sample, which limited the models’ ability to learn stable and discriminative patterns for severity stratification during training and validation (30).

**Table 4:** Comparison of three-category model performance for anxiety dimension.

Assessment model	F1 Value	Precision	Recall
SAS vs. Gold Standard	0.257	**0.312**	0.271
AL_Anx vs. Gold Standard	**0.318**	0.308	**0.432**

Bold values indicate the best result for the metric.

#### 3.2.2 Performance in risk screening

For binary anxiety-risk screening, SAS and AI-Anx performed similarly when used alone, with F1 scores of 0.471 and 0.455, respectively (**Table 5**). In contrast, a combined strategy yielded a complementary effect: recall increased from 46.2% with SAS alone to 69.2%, a 50.0% relative improvement; the F1 score also rose to 0.590, a 25.4% relative gain. These results indicate that, for anxiety screening, AI and SAS are not competing tools but rather appear to capture complementary facets of the heterogeneous manifestations of anxiety disorders (31).

**Table 5:** Binary classification model performance and combined gain for anxiety dimension.

Assessment model	F1 value	Precision	Recall	Gain effect
SAS	0.471	0.480	0.462	–
AL_Anx	0.455	0.469	0.442	–
SAS + AL_Anx	**0.590**	**0.514**	**0.692**	F1 ↑ 25.4%, Recall ↑ 50%

Bold values indicate the best result for the metric.

#### 3.2.3 ROC curve analysis

The ROC curves for the anxiety dimension (as shown in **Figure 3** (right)) visually corroborate the advantage of the combined model. Although the curves for the single models (SAS and AI-Anx) are very close to each other, the curve representing their combination is clearly elevated, with an AUC exceeding that of either individual model. This indicates that, at a given false-positive rate, the combined model attains a higher true-positive rate, thereby improving overall discrimination (27). .

#### 3.2.4 Confusion matrix analysis and clinical interpretation

**Figure 4:**
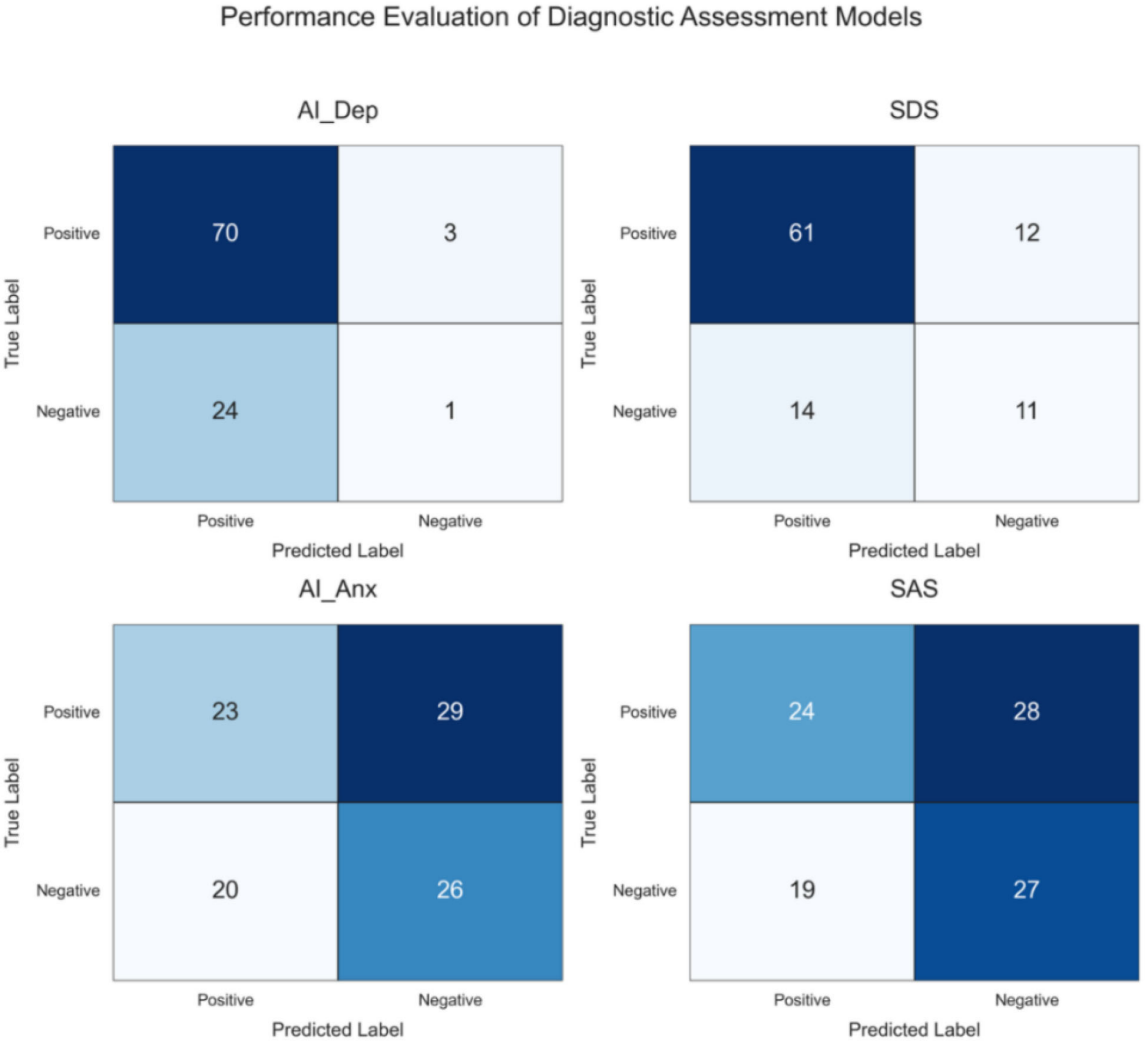
The figure presents four confusion matrices, each illustrating the classification results for a different diagnostic model: (top left) the classification results for the AI Dep model in depression screening. (top right) the classification results for the Self Rating Depression Scale (SDS). (bottom left) the classification results for the AI Anx model in anxiety screening. (bottom right) the classification results for the Self Rating Anxiety Scale (SAS).

Analysis of the confusion matrix (as shown in **Figure 4**) for the anxiety dimension further elucidated the mechanism underlying complementarity. Among 52 individuals classified as at risk for anxiety by the clinical diagnosis, AI-Anx uniquely identified 12 cases (23.1%) missed by SAS, whereas SAS uniquely identified 13 cases (25.0%) missed by AI-Anx.

A retrospective review of clinical features in these two subgroups revealed an instructive pattern: Patients uniquely identified by AI-Anx were more often described with somatic and autonomic dysregulation features, for example, palpitations, a sense of pounding heart, muscle tension and soreness, restlessness, tachypnea, and tremor (32). Their complaints focused on bodily discomfort rather than internal worry. In contrast, the SAS group more frequently reported cognitive symptoms like difficulty controlling worry or impaired concentration. These patients were able to describe their worries, but physical symptoms were less obvious (33).

These findings suggest that the AI tool (AI-Anx) may be more attuned to the “physiologic-behavioral arousal” dimension of anxiety, whereas the self-report scale (SAS) is more sensitive to the “cognitive-subjective distress” dimension. Given the inherently heterogeneous spectrum of anxiety disorders, this division of strengths explains why combining objective and subjective assessments can provide broader coverage of anxiety presentations and potentially help reduce missed diagnoses.

## 4 Discussion

In a rigorously designed diagnostic study, we conducted the first systematic domestic comparison of an AI tool based on head-neck micro-vibration analysis with internationally used self-report scales for screening depression and anxiety, and we further elucidated the mechanisms of complementarity and the added value of their combined use.

### 4.1 Paradigmatic complementarity between objective physiological markers and subjective symptom reports

The central finding of this study is that AI-based assessment and scale-based assessment embody two distinct, complementary paradigms. The AI tool’s principal advantage derives from its direct measurement of downstream outputs along the vestibulo-emotional physiological pathway. This objectivity may help bypass the inherent limitations of self-reporting. Consequently, it could aid in identifying ‘silent patients,’ whose subjective disclosure differs from their objective distress due to factors like stigma or alexithymia (25). The 15.1% of independently detected cases in the depression domain observed here attests to this strength. Conversely, because the SDS and SAS items are directly grounded in the symptom criteria of DSM-5, these instruments have a natural advantage in quantifying the presence/absence, frequency, and intensity of specific symptoms, which aligns more closely with clinicians’ reasoning in tasks that map directly onto diagnostic standards—namely, severity grading. The AI tool focuses on state detection, while scales specialize in symptom characterization. This functional distinction likely underpins the synergy observed in the combined model.

### 4.2 Capturing heterogeneity in the anxiety dimension and the need formultidimensional assessment

In the anxiety dimension, an important insight from this study is that AI and SAS appear sensitive to different facets of anxiety disorders. AI-Anx tends to identify patients whose predominant features are somatic anxiety (e.g., autonomic hyperarousal, psychomotor agitation), whereas SAS is more adept at detecting psychic anxiety (e.g., excessive worry, anticipatory fear). Anxiety disorders are inherently heterogeneous and are often divided into subtypes (e.g., generalized anxiety disorder, panic disorder) with varying contributions of physiological and cognitive components. Our findings suggest that single-scale screening might overlook patients who present primarily with somatic symptoms. These are often ‘somatized’ cases where the subjective experience of anxiety is masked. Incorporating an AI tool that captures physiological arousal signals can therefore provide broader and more dimensional coverage of the anxiety spectrum, which is crucial for improving the completeness of population-level mental health screening.

### 4.3 Exploration of a clinical screening pathway: building a “broad-screening to fine-judgment” collaborative model

Based on the above findings, we propose an optimized clinical screening pathway: “AI for broad initial screening, scales for fine-grained adjudication.” In this model, the AI tool serves as a first-line, high-sensitivity filter to conduct rapid, low-cost, and noninvasive population screening in settings such as primary care clinics, health examination centers, and schools, with the goal of maximizing case capture and quickly narrowing the pool of individuals requiring follow-up (34–36). Subsequently, standardized scales (e.g., SDS/SAS) are administered to those who screen positive on AI. This step enables finer analysis of symptom dimensions and preliminary severity grading, and it also provides cross-validation to help rule out a subset of potential AI-driven false positives. Finally, psychiatrists integrate the AI-derived objective physiologic risk signals with the detailed subjective symptom reports from the scales to make more efficient and information-rich clinical decisions (37–39). Such a layered, stepwise screening-and-intervention workflow has the potential to substantially increase early detection of psychiatric disorders without unduly burdening existing health systems, while directing scarce specialty resources more precisely to those most in need.

### 4.4 Limitations

Several limitations should be noted. First, the single-center design and modest sample size may constrain the generalizability of our findings. Second, as the participant population was skewed toward adolescents (reflecting our hospital’s patient profile), the tool’s performance in older adults and general community populations remains to be verified. Third, the exclusion of patients unable to maintain stillness limits the tool’s current applicability for individuals with severe psychomotor agitation. Finally, while the proposed workflow is promising, its feasibility in diverse clinical routines requires validation through larger, multi-center trials.

## 5 Conclusions

Through rigorous empirical analyses, this study suggests that the AI-based tool holds promise for screening depressive and anxiety disorders. It appears particularly useful for enhancing sensitivity and detecting individuals less responsive to subjective reporting. The relationship between this AI tool and traditional self-report scales (40) is not one of simple competition or substitution; rather, they are clearly and meaningfully complementary, reflecting distinct underlying mechanisms (physiologic vs. subjective). Advancing a collaborative model, in which the AI tool functions as a high-sensitivity sentinel for broad screening and self-report scales serve to refine judgments, represents a highly promising direction for early identification of psychiatric disorders. Subject to further external validation, this “objective-subjective integration” paradigm holds potential to address shortcomings in screening systems, reduce missed diagnoses, and provide a preliminary theoretical and practical foundation for building a more efficient, objective, equitable, and scalable digital public mental health infrastructure.

## Data Availability

The raw data supporting the conclusions of this article will be made available by the authors, without undue reservation.
